# IgG in the control of FcεRI activation: a battle on multiple fronts

**DOI:** 10.3389/fimmu.2023.1339171

**Published:** 2024-01-11

**Authors:** Federico Storni, Monique Vogel, Martin F. Bachmann, Paul Engeroff

**Affiliations:** ^1^ Department of Visceral Surgery and Medicine, Inselspital, Bern University Hospital, University of Bern, Bern, Switzerland; ^2^ Department of BioMedical Research, University of Bern, Bern, Switzerland; ^3^ Department of Rheumatology and Immunology, University Hospital Bern, Bern, Switzerland

**Keywords:** allergy, IgE, mast cells, basophils, degranulation, FcγRIIB, antibody, sensitization

## Abstract

The rising global incidence of IgE-mediated allergic reactions poses a significant challenge to the quality of life of affected individuals and to healthcare systems, with current treatments being limited in effectiveness, safety, and disease-modifying capabilities. IgE acts by sensitizing the high-affinity IgE receptor FcεRI expressed by mast cells and basophils, tuning these cells for inflammatory degranulation in response to future allergen encounters. In recent years, IgG has emerged as an essential negative regulator of IgE-dependent allergic inflammation. Mechanistically, studies have proposed different pathways by which IgG can interfere with the activation of IgE-mediated inflammation. Here, we briefly summarize the major proposed mechanisms of action by which IgG controls the IgE-FcεRI inflammatory axis and how those mechanisms are currently applied as therapeutic interventions for IgE-mediated inflammation.

## 1 FcεRI sensitization and activation

Whether IgE switching occurs from IgM or IgG remains a debated topic and has been discussed elsewhere (1, 2). Here, we focus on how IgG controls the immediate allergic reaction once IgE is produced by B cells. The activation of inflammatory mast cell and basophil degranulation by IgE is a two-step process (3, 4). First, IgE needs to sensitize mast cells and basophils by binding to the high-affinity IgE receptor FcεRI. This interaction is remarkably stable and sensitization can thus persist for long periods, specifically in the more long-lived mast cells (5, 6). The second event is the encounter with an antigen that cross-links IgE bound to FcεRI leading to FcεRI intracellular signaling cascades and degranulation (7, 8). If improperly controlled, this reaction can have negative consequences such as in allergy, where harmless antigens (allergens) become activators of FcεRI. Currently, allergic diseases are posing massive challenges to global health and thus, strategies to inhibit the IgE : FcεRI inflammatory axis have been of high interest to clinical and pharmaceutical research (9, 10). In recent years, it has become clear that IgG antibodies may be important in both types of physiological FcεRI de-granulation control, the sensitization and the activation phase.

## 2 IgG specific for IgE in the prevention of FcεRI sensitization

We and others have shown that naturally occurring IgG anti-IgE autoantibodies are present to surprising levels in humans and mice. In both humans and mice, the antibodies can prevent basophil sensitization and FcεRI activation (11–14). Interestingly, the immunogenicity of self-IgE is regulated by a single highly conserved mannose glycosylation site on IgE, which is likewise preferentially recognized by the anti-IgE autoantibodies (12, 15). The same mannose glycan is crucial for both FcεRI binding and IgE sensitization (16, 17). It thus seems plausible that competition between anti-IgE autoantibodies and FcεRI for this single mannose glycosylation site explains how IgE-IgG ICs fail to activate FcεRI. Nevertheless, this aspect remains to be studied in further detail. We have shown another mechanism by which anti-IgE auto-antibodies act. The formation of IgG immune complexes with IgE (IgE-IgG-IC) results in preferential targeting of the low-affinity IgE receptor CD23 over FcεRI (12). CD23 is constitutively expressed in B cells but can be induced in a variety of other cells (18). We had previously shown that CD23 is essential in the clearance of IgE-allergen-IC but this is likewise the case for IgE-IgG-ICs (12, 19). Moreover, the immune response caused by IgE-IgG-ICs results in a boost of anti-IgE autoantibodies establishing a positive feedback loop that diminishes serum IgE levels further. Interestingly, studies have proposed maternal transfer of IgE-IgG-IC via the neonatal FcRn receptor (20, 21). It is important to note, that any dysregulation of those anti-IgE autoantibodies may lead to pathologies, for example they are thought to inadvertently cross-link FcεRI in Chronic Spontaneous Urticaria (CSU) or atopic dermatitis (AD) thus contributing to disease (22, 23). Overall, the role of natural anti-IgE antibodies in health and disease needs to be further investigated in future studies.

## 3 IgG specific for allergen in the inhibition of FcεRI activation

Once IgE has bound to FcεRI, allergen-specific IgG may still block allergic reactions by allergen-neutralization. IgG circulates the blood at very high concentrations compared to IgE and if enough specific IgG is present, is thus likely to form IgG-allergen ICs before FcεRI is encountered. Numerous studies have shown a role for blocking antibodies in preventing the initiation of the allergic cascade by impeding the binding of allergens to IgE (24–26). Due to their lower ability to activate complement and Fc-dependent effects, IgG4 antibodies have been considered specifically optimal blocking antibodies in allergy (27–30). However, neutralizing IgGs are not required to suppress FcεRI. Even if an IgG-allergen IC reaches the cell and is recognized by IgE, there is a second mechanism at work that suppresses the allergic response. Mast cells and basophils can express the inhibitory IgG receptor FcγRIIb, which contains an ITIM motif that activates the inositol phosphatase SHIP, a major negative immune regulator (31, 32). When FcεRI is cross-linked to FcγRIIb, the activation cascade of FcεRI is suppressed via SHIP (32–35). Notably, the inhibitory signal from FcγRIIb does not only act on individual allergen-IgG pairs but SHIP can spread around the cell membrane and dephosphorylate distant FcεRI and activated kinases (36). While it was often speculated that IgG4 is superior in suppressing the allergic reaction via FcγRIIb, it was recently shown that IgG1 and IgG4 appear to similarly suppress FcεRI activation via FcγRIIb (33, 37). Moreover, IgG affinity for the allergen seems less important for FcγRIIb engagement than for neutralizing antibodies (38). Finally, FcεRI : FcγRIIb complexes can be internalized and degraded, resulting in a de-sensitizing of effector cells, coupled with a reduction of IgE (39). In actuality, both neutralization and FcγRIIb engagement most likely occur simultaneously during polyclonal responses *in vivo* (40).

## 4 The role of IgG in allergy immunotherapy

Elevated levels of IgG antibodies have been observed in individuals with natural tolerance to allergens, suggesting a possibly inherent protective role of these antibodies (41). In mice, maternal IgG-allergen complexes transferred via breast milk and the neonatal receptor FcRn were shown to protect the offspring from allergy (42). On the other hand, IgG has been extensively studied in allergen-specific immunotherapy (AIT), a disease-modifying treatment for allergy, where increasing doses of specific allergens are administered to allergic individuals to build tolerance (43, 44). Successful AIT has been associated with elevated levels of allergen-specific IgG. Specifically, it is accepted that the induction of high IgG : IgE ratios in addition to regulatory T and B cells is a favorable and protective occurrence whereas IgE levels are initially increased with AIT before they are later reduced (30, 45–49). Moreover, high IgG levels have been associated with reduced risk of more severe, anaphylactic reactions (50). While in AIT, the IgG4 subclass is usually associated best with protection from allergy, this most likely reflects superior induction of IgG4 over IgG1 by most AIT-protocols and does not necessarily indicate an inferiority of IgG1 to neutralize the allergen or engage FcγRIIb. Recently, it was shown that IgG1 may suppress the allergic during earlier AIT responses whereas IgG4 gains importance in later stages (37, 49). Even though some mechanistic aspects remain elusive, current findings suggest AIT, the only current disease-modifying therapy for allergic diseases involves the induction of protective IgG antibodies.

## 5 Therapeutic approaches based on the anti-allergic properties of IgG

The IgG-mediated control of FcεRI sensitization and activation have been increasingly translated into clinical development and application. Omalizumab, a monoclonal anti-IgE antibody which blocks FcεRI sensitization has been developed a long time ago (51). Mechanistically, anti-IgE biologics act similar to natural anti-IgE autoantibodies by preventing or disrupting the FcεRI-IgE interaction and by lowering serum IgE levels. We have recently shown that omalizumab function, in identical fashion to natural anti-IgE autoantibodies, depends on the single IgE mannose glycosylation site (52). A number of other IgE-targeting approaches are explored, which have been reviewed by others (53–61). The development of allergen-specific IgG for the treatment of allergy is a more recent development and for the most part, clinical trials are still ongoing (62, 63). Two major strategies are currently being employed, both aiming at harnessing the protective role of allergen-specific IgG. Neutralizing monoclonal anti-allergen IgG antibodies have demonstrated efficacy in suppressing allergy in preclinical and clinical settings (64–72). While the results are generally positive, questions on their cost-effectiveness remain. Nevertheless, their ability to increase the safety of allergy immunotherapy and potentially boost beneficial immune responses is intriguing (63). A more cost-effective approach could be the induction of polyclonal IgG responses using immunization strategies that improve the current drawbacks of AIT including safety concerns, patient compliance, long treatment periods and varying efficacy. The challenge here is to modify the allergens in a way that reduces FcεRI cross-linking but enhances the induction of allergen-specific IgG. Others have reviewed the current landscape of vaccination strategies for improving AIT (73–79). We have focused on using allergens displayed on virus-like particles (VLP), which can reduce allergen reactivity while boosting IgG responses that increase protection via FcγRIIb (80, 81).

## 6 Discussion: future prospects and challenges

**Figure 1:**
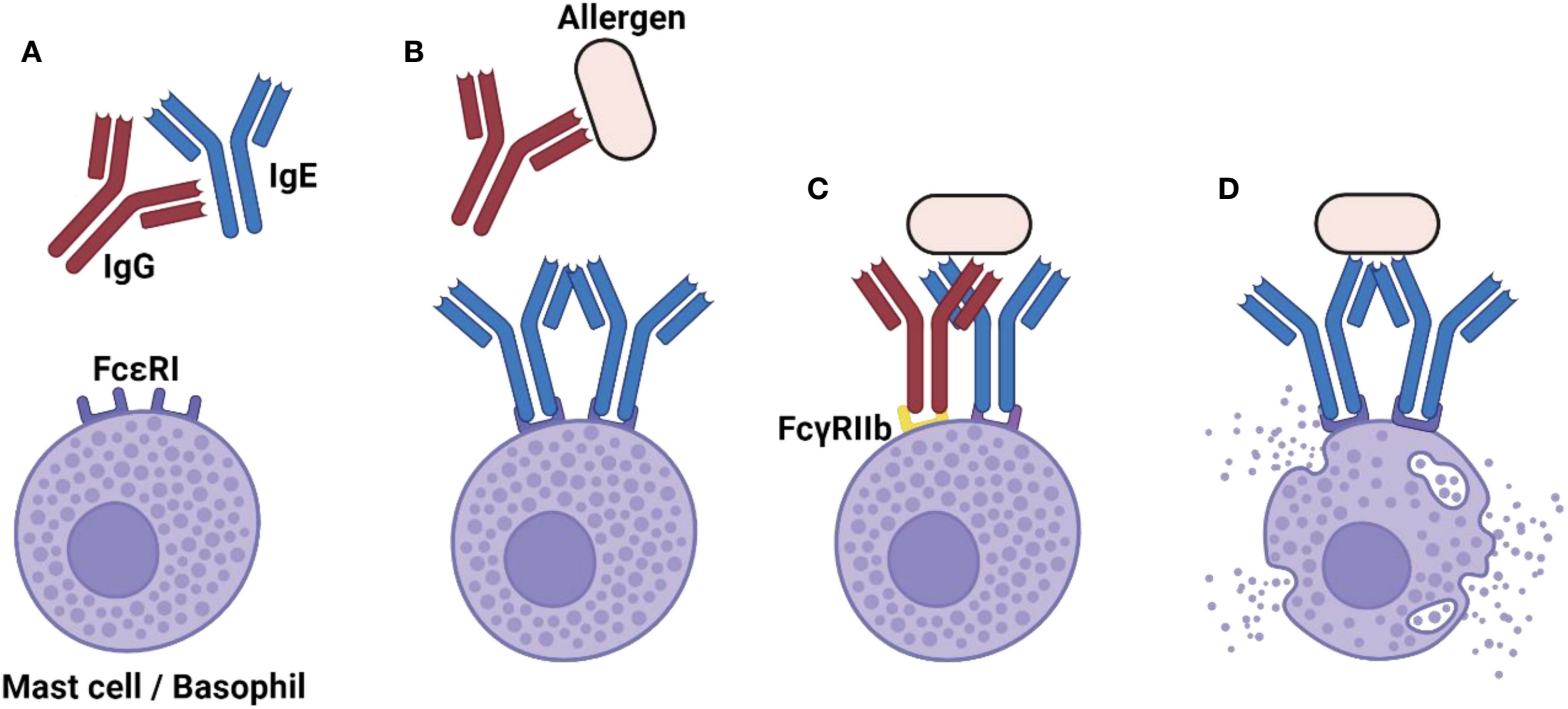
The diverse role of IgG in shutting down-the allergic reaction. **(A)** Naturally occurring IgG anti-IgE autoantibodies can limit the extent of FcεRI sensitization and thereby prevent the allergic reaction. **(B)** In the case that FcεRI sensitization has already occurred, neutralizing IgG anti-allergen antibodies can block allergen recognition by IgE, thus preventing FcεRI activation. **(C)** Even in absence of neutralizing IgG antibodies, the inhibitory FcγRIIb receptor suppresses the allergic reaction when it is cross-linked to FcεRI. **(D)** In the absence of IgG-mediated control, the allergic reaction can occur. Created with BioRender.com.

The overall importance of IgG in the control of IgE : FcεRI in basic and preclinical research is established. Ongoing and future research will define the relative importance of the here-presented individual pathways through which IgG confers protection against allergies (**Figure 1**). As research advances, it is expected that a more nuanced and contextual understanding of IgG in allergic reactions will be achieved. This will pave the way for innovative therapeutic strategies translating the protective potential of IgG into the clinics, thereby offering relief to millions of people suffering from allergies worldwide.

## Author contributions

FS: Writing – original draft. MV: Writing – review & editing. MB: Writing – review & editing. PE: Writing – original draft.
